# Systemic Sclerosis: From Pathophysiology to Novel Therapeutic Approaches

**DOI:** 10.3390/biomedicines10010163

**Published:** 2022-01-12

**Authors:** Devis Benfaremo, Silvia Svegliati, Chiara Paolini, Silvia Agarbati, Gianluca Moroncini

**Affiliations:** 1 Clinica Medica, Department of Internal Medicine, Ospedali Riuniti “Umberto I-G.M. Lancisi-G. Salesi”, 60126 Ancona, Italy; d.benfaremo@pm.univpm.it; 2 Department of Clinical and Molecular Sciences, Marche Polytechnic University, 60126 Ancona, Italy; s.svegliati@staff.univpm.it (S.S.); c.paolini@univpm.it (C.P.); s.agarbati@staff.univpm.it (S.A.)

**Keywords:** systemic sclerosis, pathophysiology, therapy

## Abstract

Systemic sclerosis (SSc) is a systemic, immune-mediated chronic disorder characterized by small vessel alterations and progressive fibrosis of the skin and internal organs. The combination of a predisposing genetic background and triggering factors that causes a persistent activation of immune system at microvascular and tissue level is thought to be the pathogenetic driver of SSc. Endothelial alterations with subsequent myofibroblast activation, excessive extracellular matrix (ECM) deposition, and unrestrained tissue fibrosis are the pathogenetic steps responsible for the clinical manifestations of this disease, which can be highly heterogeneous according to the different entity of each pathogenic step in individual subjects. Although substantial progress has been made in the management of SSc in recent years, disease-modifying therapies are still lacking. Several molecular pathways involved in SSc pathogenesis are currently under evaluation as possible therapeutic targets in clinical trials. These include drugs targeting fibrotic and metabolic pathways (e.g., TGF-β, autotaxin/LPA, melanocortin, and mTOR), as well as molecules and cells involved in the persistent activation of the immune system (e.g., IL4/IL13, IL23, JAK/STAT, B cells, and plasma cells). In this review, we provide an overview of the most promising therapeutic targets that could improve the future clinical management of SSc.

## 1. Introduction

Scleroderma, or systemic sclerosis (SSc), is an immune-mediated chronic disorder with a systemic involvement characterized by small vessel alterations and progressive fibrosis of the skin and internal organs, such as lungs, gastrointestinal tract, and heart [1]. SSc is a devastating and severe disease, burdened with significant morbidity and mortality. When a severe pulmonary or cardiac involvement is present, patients with SSc have a 3-year survival rate of 47–56% [2,3,4].

The skin is involved in the majority of patients with SSc. Two different clinical subsets, according to the extent of skin involvement, are usually recognized: diffuse cutaneous SSc (dcSSc), in which the skin damage is extended proximally to elbows and/or knees or to thorax and/or abdomen at any given time during disease course, and limited cutaneous SSc (lcSSc), in which the skin damage remains distal to elbows and knees without involvement of either thorax or abdomen. Although this clinical distinction has been demonstrated to be able to predict the onset of severe complications, including life-threatening lung and heart fibrosis, classification based on the extent of skin involvement has several limitations [5].

Scleroderma is now conceived as a complex syndrome with multiple pathogenic pathways. While there are still many unanswered questions, understanding of these pathways has greatly improved in recent years. In particular, the central role of immune system cells and inflammatory mediators, fibroblasts, and other cells determining the regulation of the extracellular matrix (ECM) is now recognized [6,7].

Substantial progress has also been made in the management of SSc complications in recent years, which has led to increased survival and quality of life. This includes better control of complications in specific organs (such as interstitial lung disease (ILD) [8], pulmonary arterial hypertension (PAH) [9], scleroderma renal crisis, and Raynaud’s phenomenon), as well as standardized follow-up and early diagnosis of potential complications [10].

Despite recent advances, however, an effective disease-modifying treatment approved for the treatment of SSc is currently lacking. Given the heterogeneity of pathways implicated in SSc onset and progression, new treatment strategies for SSc should ideally target many, if not all, such pathogenetic pathways, including those involved in immune activation, immune-mediated inflammation, vasculopathy, and fibrosis.

Herein, we review the pathogenic pathways, current treatment, and potential new therapeutic approaches for SSc.

## 2. Pathogenic Pathways in Systemic Sclerosis

**Figure 1:**
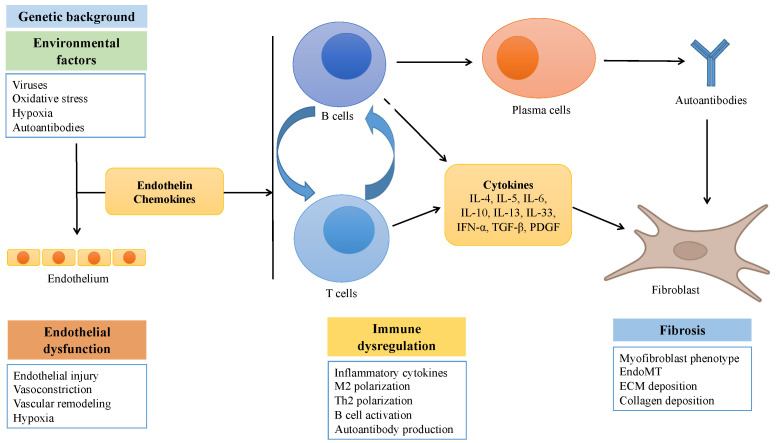
Simplified scheme showing the main processes involved in the pathogenesis of systemic sclerosis.

The etiopathogenesis of SSc is still elusive. As for many other immune-mediated diseases, the most accepted hypothesis is that the combination of a predisposing genetic background and a triggering factor or event may cause a break of tolerance toward self-antigens with persistent activation of the immune system, not timely downregulated by endogenous regulatory cells and checkpoints. The first site of persistent immune-mediated inflammation is most likely the microvessel wall, causing alterations involving not only the endothelium, but also all the vessel layers, with subsequent myofibroblast activation, excessive extracellular matrix (ECM) deposition, and unrestrained tissue fibrosis [11] (Figure 1).

Several genetic loci have been associated to increased risk of developing SSc, including genes encoding molecules specifically involved in fibrotic and/or vasculopathy pathways [12,13,14]. Recently, few HLA class II and one HLA class I alleles were found to be strongly enriched in the SSc cohort and, of note, selectively associated with distinct clinical and serological SSc subsets, providing novel functional insights [15].

### 2.1. Immunological Changes

The latter report highlights, once more, the role of the immune system in SSc. Dysregulation of the immune system is witnessed by the presence of autoantibodies, several of which are exclusive of this disease and associated with clinical complications and specific phenotypes (Table 1) [6,16]. Some of these autoantibodies have been directly implicated in the pathogenesis of SSc. For example, agonistic antibodies stimulating the Platelet Derived Growing Factor Receptor α (PDGFRα) are capable of inducing the persistent activation of the intracellular signaling cascade that is usually transiently triggered by PDGF, leading to chronic myofibroblast activation and subsequent ECM accumulation [16,17,18]. Unlike the natural, non-stimulatory, anti-PDGFRα autoantibodies, agonistic anti-PDGFRα autoantibodies recognize specific conformational epitopes, largely overlapping with the PDGF binding site, suggesting their pathogenic role in the SSc-specific, unbalanced autoimmune response against cellular antigens [19,20,21].

B cells from SSc patients not only produce autoantibodies, but can also infiltrate tissues and show increased activation markers such as CD19, CD21, costimulatory molecules, and B cell activating factor (BAFF). There is evidence in murine models that overexpression of CD19 induces the production of cutaneous fibrosis and that the absence of B cells is associated with decreased fibrosis [14].

Moreover, T lymphocytes from SSc patients show an increased expression of activation markers. Th2 cells, producing IL-4 and IL-13, and Th17, and producing IL-17, are increased in both skin and peripheral blood of patients with SSc, particularly in patients with the diffuse form of the disease [22]. The role of regulatory T cells in SSc is less clear [23]. Other relevant immune system cells, such as macrophages and dendritic cells (DCs), can also infiltrate skin of SSc patients, producing proinflammatory and profibrotic cytokines and chemokines, which in turn are responsible for the process of endothelial-mesenchymal transition (EMT). In this process, endothelial and epithelial cells are activated and acquire characteristics similar to myofibroblasts [24]. Fibrocytes from the peripheral circulation are also co-opted to become activated fibroblasts producing collagen and other ECM proteins [11].

Although the factors that promote the persistent activation of cells of the immune system are unknown, recent studies have highlighted the possible role of Toll-like receptors (TLR) in the activation of dendritic cells, which could in turn secrete proinflammatory cytokines and present antigens to the T cells. Overexpression of TLR4 and TLR2 has been found in skin and fibroblasts of patients with SSc [25,26]. Activation of dendritic cells through TLRs generally leads to the production of several proinflammatory cytokines, particularly type I interferons (IFN), which have been found overexpressed in the sera of patients with SSc [27]. Up to 50% of SSc patients may show this so-called “interferon signature” in the peripheral blood. Interestingly, these abnormalities may be seen in early phases of the disease, before overt skin fibrosis [27]. The increased expression of type I IFN in SSc may induce monocyte activation, as well as increased differentiation, survival, proliferation, and activation of T, B, and dendritic cells [28]. Moreover, type I IFN stimulates the expression of TLRs on DCs and fibroblasts, indirectly leading to increased inflammatory cytokine production by fibroblasts.

Interleukin (IL)-33 is an alarmin of the IL-1 family related to inflammation and fibrosis and has been recently implicated in the pathogenesis of SSc [29]. Following the production of IL-33 and IL-25 by innate lymphoid cells, there is an overproduction of IL-4 and IL-13 that increases collagen deposition by fibroblasts and induces the differentiation of macrophages towards a profibrotic phenotype [30]. Evidence of the involvement of macrophages in the pathogenesis of SSc is extensive. Increased CD14+CD163+CD204+ cells have been found in peripheral blood of patients with SSc, as well as increased markers of macrophage migration and activation (CCL18 and CD163) in microarrays of lung tissue from patients with progressive pulmonary fibrosis [31]. Macrophages can also be stimulated through TLRs and their activation towards a profibrotic phenotype (M2 polarization) would lead to the production of cytokines such as IL-6, IL-10, and IL-13, transforming growth factor (TGF)-β and PDGF [32,33].

### 2.2. Endothelial Dysfunction

The endothelium plays a pivotal role in the initiation and perpetuation of vasculopathy associated with SSc. Endothelial damage is thought to occur early in the pathogenesis of SSc. The activation of endothelial cells leads to vasoconstriction and subendothelial fibrosis, contributing to the development of intraluminal thrombosis and proliferation of the muscular layers [34], which in turn leads to the vascular phenomena of SSc. Abnormal angiogenesis due to the increased expression of angiogenic factors such as PDGF, VEGF, and ET-1, and TGF-β is also a hallmark of SSc [35].

### 2.3. Fibrotic Changes

Fibrosis, consequent to the excessive production of collagen and other ECM proteins, is the key pathogenic alteration of SSc and the main process that leads to end-organ failure [11]. Myofibroblasts can derive from different type of cells, both resident and circulating endothelial cells, following EMT [34]. The aberrant activation of endothelial cells leads in turn to expression of alpha smooth muscle actin (αSMA), vimentin, and type I collagen, until they become similar to myofibroblasts [36].

Additionally, in patients with SSc there is evidence of abnormal epithelial regeneration, leading to EMT via the upregulation of ET-1 and TGF-β [37]. During EMT, epithelial cells lose their intercellular junctions, change their polarity and express different surface markers, gradually gaining a mesenchymal phenotype [38].

Depending on the microenvironment, fibroblasts can produce different amounts of procollagen, fibronectin, proteases, collagenases, and other regulators of the extracellular matrix. For example, inactive fibroblasts express ET-1 and ICAM-1, whereas fibroblasts exposed to mechanical stress in the microenvironment, a situation that occurs in SSc, express α-SMA, TGF-β, and genes associated with the production of ECM proteins [39,40].

## 3. Current Therapeutic Options in Systemic Sclerosis

There are several treatment options for managing the diverse clinical manifestations of SSc (Table 2, [41]).

### 3.1. Vasodilating Agents

Vasodilators, such as calcium antagonists, phosphodiesterase type 5 (PDE5) inhibitors, and synthetic analogues of prostacyclin and endothelin receptor antagonists (ERAs), are indicated for the treatment of Raynaud phenomenon, ischemic digital ulcers, and PAH.

Bosentan, ambrisentan, and macitentan are the endothelin receptor antagonists currently approved for the treatment of PAH, including connective tissue disease associated-PAH.

Bosentan has higher affinity towards ETB receptors and essentially the same affinity for the ETA receptors, and it occupies the orthosteric site of the receptor to block the action of ET-1 by sterically preventing the inward movement of transmembrane helix six of the ETB receptor [42], a mechanism that is expected to be preserved in the ETA subtype. Aside from PAH, it has also been proven to reduce the number of new digital ulcers, even in patients with multiple ones, regardless of usage of calcium channel blockers, PDE-5 inhibitors, or iloprost therapy, having a highly evident effect in patients with four or more digital ulcers at baseline in the RAPIDS-2 trial [43].

On the other hand, macitentan was designed to have improved efficacy and higher potency and selectivity over bosentan [44]. Additionally, macitentan and its active metabolite both have a long half-life (16 and 48 h, respectively), which supports a once-daily dosing regimen. It has been approved for the long-term treatment of patients with PAH as monotherapy or in combination with other therapies, following the results of the SERAPHIN trial [45]. Given that bosentan has previously been shown to prevent the occurrence of new digital ulcers in patients with SSc, even though it had no effect on their healing [43], the results of the DUAL trial, in which macitentan showed no efficacy in reducing the burden of digital ulcers in SSc patients, were quite disappointing [46].

Oral phosphodiesterase 5 (PDE-5) inhibitors include sildenafil and tadalafil [47]. Both agents are approved for the treatment of PAH, having showed remarkable efficacy alone or in combination with ERAs [41]. Moreover, the efficacy of sildenafil in healing digital ulcers has been demonstrated in the SEDUCE trial [48]. PDE5 inhibitors prevent the hydrolysis of cGMP, which has vasodilatory and antiproliferative effects on the pulmonary vasculature [48].

Riociguat is a soluble guanylate cyclase (sGC) stimulator with vasoactive, anti-proliferative and anti-fibrotic effects. It is currently approved for the treatment of connective tissue disease (CTD)-associated PAH following the results of the PATENT studies [49]. In a recent trial, treatment with riociguat did not reduce the number of digital ulcers compared to the placebo at 16 weeks [50]. Despite its well-known action on fibrosis in vitro [51], riociguat has disappointed the expectations in the recently reported phase IIb RISE-SSc study, where the primary endpoint was not met even though there was a difference in the skin score progression rate favoring riociguat over placebo at week 52 [52].

Selexipag is an oral, selective IP prostacyclin receptor agonist that has recently been approved for the long-term treatment of PAH [53]. Selexipag is considered to have a good safety profile, with minimal adverse events, ranging from mild to moderate in severity [54]. In a post hoc analysis of the GRIPHON trial, selexipag reduced the risk of composite morbidity/mortality events in patients with CTD-PAH by 41% [55]. Given its mechanism of action, selexipag treatment was unexpectedly shown to be no better than placebo for the prevention and treatment of Raynaud’s phenomenon attacks in patients with SSc [56].

### 3.2. Immunosuppressive Agents

Immunosuppressive agents, such as methotrexate (MTX), cyclophosphamide (CYC), and mycophenolate mofetil (MMF), are indicated for skin disease and ILD.

MTX had previously shown some efficacy on skin disease in small non-recent studies [57,58].

The efficacy of CYC in SSc has been extensively evaluated in a phase III trial (Scleroderma Lung Study I), in which it demonstrated a significant but modest beneficial effect on lung function, dyspnea, thickening of the skin, and the health-related quality of life. Though modest, the effects on lung function were maintained through the 24 months of the study [59]. More recently, the potential efficacy of CYC on both skin and lung fibrosis has also been reported by several observational studies [60,61].

In the Scleroderma Lung Study II, treatment of SSc-ILD with MMF for 2 years, or CYC for 1 year, both resulted in significant improvements in lung function over the two-year course of the study. Given the better tolerability and toxicity profile, MMF is currently the preferred treatment for SSc-ILD [62]. Post hoc analyses from SLS-II also demonstrated that both MMF and CYC treatment resulted in improvements in skin disease in patients with dcSSc over 24 months [63].

Based on the results of a large phase III trial [64], in which it showed long-term benefits in patients with SSc, including improved event-free and overall survival, at a cost of increased toxicity, autologous hematopoietic stem-cell transplantation (HSCT) has also been recently recommended for the treatment of patients with progressive disease who are at risk of organ failure.

Abatacept is a CTLA-4-Ig fusion protein that inhibits the CD80/86-CD28 costimulatory pathway preventing T cell activation. Although abatacept is not approved for the treatment of SSc, a recent phase II study, and its open-label extension, showed that a clinically significant improvement of skin sclerosis and disability could be achieved with abatacept over an 18-month period, with a good safety profile, albeit the study did not meet its primary endpoint [65].

Rituximab (RTX) is a well-known chimeric monoclonal antibody targeting CD20, which is extensively expressed from the pre-B cell stage to the pre-plasma cell stage [66]. Given its mechanism and the potential role of B cell depletion for the treatment of SSc, as outlined in the previous sections, several observational studies that used off-label RTX for the treatment of SSc patients were conducted, suggesting its antifibrotic effect, as well as its potential to improve inflammatory alterations and lung function [67,68,69,70].

In a recent systematic review [71] that included three RCTs and five cohort studies, RTX was associated with a significant improvement of the skin score and, only in RTCs, of lung function. As such, RTX may be an alternative treatment for cutaneous and pulmonary manifestations in patients with SSc, with a favorable safety profile.

Tocilizumab (TCZ) is a monoclonal antibody directed against the IL-6 receptor that has been recently evaluated for the treatment of SSc. IL-6 is thought to play a prominent role in the pathogenesis of SSc [72]. Indeed, there is an increase in IL-6 expression in endothelial cells and skin fibroblasts and an increase in serum IL-6 levels in SSc patients [73,74].

In the phase II study (faSScinate), TCZ was not associated with a significant reduction in skin thickening. However, less decline in FVC was observed in SSc patients treated with TCZ versus placebo [75]. Results from the extension study suggested that patients originally assigned to receive placebo in the double-blind period who transitioned to open-label TCZ at week 48 experienced improvements in the skin thickness by week 96 that were similar to those of patients who received TCZ throughout the study. Furthermore, patients originally assigned to receive TCZ during the double-blind period maintained and continued the improvements in the skin score observed during the first 48 weeks of treatment on receiving another 48 weeks of open-label TCZ. Finally, no patients who completed week 96 of the study experienced a decline more than 10% in %FVC during the open-label period while receiving TCZ [76].

Although the subsequent phase III trial (focuSSced) [77], which randomized 210 SSc patients, failed to meet its primary end point (change in skin score) at week 48, patients treated with TCZ had a lower rate of decline of FVC, particularly in patients with elevated acute-phase reactants.

Post-hoc analyses of the focuSSced trial confirmed that TCZ stabilized FVC% over 48 weeks in SSc-ILD with progressive features, regardless of the radiographical extent [78].

Considering the lack of effective therapies in SSc, TCZ should be considered for the treatment of SSc-ILD, especially for patients at high risk of progression [79].

### 3.3. Anti-Fibrotic Agents

The most recent additions to the therapeutic armamentarium available for SSc are agents targeting the fibrotic pathway. Nintedanib, a tyrosine kinase inhibitor (TKI) targeting fibroblast growth factor (FGF) receptor, PDGF receptor, and vascular endothelial growth factor (VEGF) receptor, has been recently approved for the treatment of SSc-ILD by the Food and Drug Administration (FDA) and the European Medicines Agency (EMA), following the results of the phase III SENSCIS trial [80]. In this study, among 663 patients were randomized, the adjusted rate of decline in the FVC was lower with nintedanib than with the placebo, for a between-group difference of 107.0 mL per year. Diarrhea was the most common adverse event, reported in 66.9% and 23.9% of patients treated with nintedanib and placebo, respectively. Nintedanib treatment was not effective for skin disease.

Several post-hoc and subgroup analyzes of the SENSCIS study suggest that the efficacy of nintedanib is consistent across the disease spectrum, regardless of predicted % FVC, disease duration, magnitude of fibrotic changes in HRCT, autoantibody status, SSc subtype, and drug use (including MMF) at baseline [81].

The more recent INBUILD trial, which enrolled 663 patients, including participants with SSc-ILD and progressive features (i.e., a relative decline in the FVC of at least 10% of the predicted value, a relative decline in the FVC of 5% to less than 10% of the predicted value, and worsening of respiratory symptoms or an increased extent of fibrosis on high-resolution CT, or worsening of respiratory symptoms and an increased extent of fibrosis), further confirmed the benefit of this antifibrotic in reducing the rate of decline of lung function, independent of the fibrotic pattern on high-resolution CT, though without meaningful changes in quality of life [82]. Further longitudinal studies are needed to assess the long-term efficacy of nintedanib in SSc-ILD and its impact on mortality and quality of life measures.

Current therapeutic options are mainly limited to the management of vascular and fibrotic manifestations, but none of them are actually curative or disease-modifying. Moreover, none of these therapies have been shown to substantially improve survival. Finally, some of these therapies are burdened with the occurrence of clinically important adverse events, even death, as in the case of HSCT. The development of new targets and treatment options for SSc is therefore warranted.

## 4. Novel Agents Targeting Inflammation and Fibrosis

Several molecules involved in SSc pathogenetic pathways are under evaluation as possible therapeutic targets in clinical trials, and agents targeting these pathways are in different stages of development (Table 3).

### 4.1. Agents in Phase III

Lenabasum is an oral small-molecule, selective cannabinoid receptor type 2 (CB2) agonist, which preferentially binds to CB2 expressed on activated immune cells and fibroblasts. CB2 activation triggers physiologic pathways that resolve inflammation, speed bacterial clearance, and halt fibrosis [83,84]. CB2 activation also induces the production of specialized pro-resolving lipid mediators that activate an endogenous cascade responsible for the resolution of inflammation and fibrosis, while reducing production of multiple inflammatory mediators. Through activation of CB2, lenabasum also is designed to have a direct effect on fibroblasts. Lenabasum is believed to induce resolution of inflammation rather than immunosuppression by triggering biological pathways to turn “off” chronic inflammation and fibrotic processes [85].

Lenabasum has demonstrated promising efficacy in preclinical models of inflammation and fibrosis. Preclinical and human clinical studies have shown lenabasum to have a favorable safety, tolerability, and pharmacokinetic profile. At the clinical level, the drug has demonstrated significant benefit in a phase II study in dcSSc, maintaining the improvements in skin scores and multiple patient-reported outcomes in the long-term open-label extension study [86,87].

A phase III trial of lenabasum administration in patients with diffuse SSc has been completed in December 2020 (NCT03398837).

Pirfenidone is an antifibrotic agent with anti-inflammatory properties, including inhibition of proinflammatory cytokines and inhibition of inflammatory cell proliferation [88]. Pirfenidone has been approved for the treatment of patients with idiopathic pulmonary fibrosis (IPF), a chronic, progressive, and almost invariably fatal disease [89,90]. Despite differences in their clinical presentation, IPF and SSc-ILD share some overlapping pathogenic mechanisms, including injury to structural cells, fibroblast activation, myofibroblast accumulation, expression of fibrogenic cytokines and growth factors, and progressive ILD. Pirfenidone is generally well tolerated in patients with IPF, and compared with placebo, pirfenidone significantly reduces disease progression (as measured by change in percent-predicted FVC) and increases progression-free survival. Pirfenidone also significantly reduces the risk of mortality in patients with IPF compared with placebo.

In the phase II LOTUSS study, designed to assess the safety of pirfenidone in patients with SSc-ILD at the same therapeutic dose used in IPF, the drug showed an acceptable tolerability profile but exploratory disease outcomes remained largely unchanged [91].

The Scleroderma Lung Study III, a phase III trial evaluating the combination of MMF and pirfenidone in SSc-ILD, is currently ongoing (NCT03221257). However, in a small randomized controlled trial conducted in India, treatment with pirfenidone failed to demonstrate a beneficial effect over placebo in stabilizing FVC, functional status, or skin disease after 6 months of therapy [92].

IL-23/IL-17 axis is implicated in the pathogenesis of several autoimmune and inflammatory diseases [93,94]. Although its precise role in SSc is unclear, preclinical studies indicate a potential role for IL-17 in regulating dermal and cardiac fibroblast proliferation, [95,96] and altered IL-17 expression has been reported in cells from patients with SSc [97]. Moreover, growing evidence from experimental models of fibrosis indicates that expression of IL-17 and of its receptor is upregulated in lung fibrosis, with increased expression of TGFβ [98].

The use of biologic agents that target IL-17 is therefore a promising potential strategy in SSc. A phase III clinical trial of brodalumab, an IL-17 receptor antagonist, in currently ongoing (NCT03957681).

Likewise, a phase II clinical trial evaluating the safety and efficacy of guselkumab, a IL23-p40 inhibitor, has just started recruiting (NCT04683029).

### 4.2. Agents in Phase II

Though promising, RTX alone has some limitations in the treatment of a complex disease such as SSc. Several lines of evidence indicate that the major limit may be the persistence of autoreactive long-lived plasma cells, not targeted by RTX, in various survival niches [99]. Moreover, RTX treatment triggers the secretion of B-cell activating factor (BAFF), which perpetuates autoreactive B cells [100]. To overcome these issues, different solutions have been proposed.

The first is to use belimumab, a BAFF inhibitor, currently licensed for use in systemic lupus erythematosus (SLE). In a pilot phase II trial [101], 20 patients with dcSSc were randomized to either belimumab or placebo. Although there was a greater improvement in the skin thickness in the belimumab group, the difference did not achieve statistical significance. However, changes in gene expression were consistent with mechanism of action and showed that clinical response to treatment with belimumab is associated with a significant decrease in profibrotic genes and pathways. To investigate the efficacy of the combination of RTX and belimumab in SSc, a phase II trial is currently ongoing (NCT03844061).

Another possibility to overcome the limitations of RTX in depleting antibody-producing plasma cells is to target them with proteasome inhibitors.

Anecdotal evidence shows that bortezomib, a proteasome inhibitor licensed for multiple myeloma, is able to induce a depletion of autoantibodies and control disease manifestations in patients with various autoimmune diseases, including primary Sjögren’s syndrome, refractory SLE and ANCA-associated vasculitis [99]. A small phase II trial with bortezomib in SSc has been conducted (NCT02370693), but results have not been reported yet.

Ixazomib is the first orally available proteasome inhibitor. A proof-of-concept clinical trial in SSc in ongoing (NCT04837131).

Romilkimab (SAR156597) is a humanized bispecific IgG4 antibody that utilizes an innovative tetravalent bispecific tandem immunoglobulin format to bind and neutralize circulating IL-4 and IL-13. Both IL-13 and IL-4 are important mediators in allergy and, most importantly, fibrosis. SSc is well-known to be associated with a Th2 polarization and IL-13 levels are elevated both in the blood and in the skin of patients [102]. Therefore, IL-13 may be a promising target in SSc and fibrotic disorders.

Despite these premises, a recent phase II study failed to demonstrate a benefit for romilkimab in the treatment of IPF [103]. A proof-of-concept phase II trial in diffuse SSc has recently been reported [104]. In this study, which randomized 97 patients to romilkimab or placebo for 24 weeks, change in the skin score favored romilkimab. Adverse events were mostly mild-to-moderate and discontinuations were low. The significant effects on skin changes with romilkimab in early dcSSc await confirmation in a phase III study.

Sirolimus (rapamycin) is an oral inhibitor of mammalian target of rapamycin (mTOR), acting through the reduction in protein phosphorylation, cell cycle progression, and cytokine production. It is currently approved for the prophylaxis of organ rejection in adult patients at low to moderate immunological risk receiving a renal transplant.

Sirolimus was demonstrated to inhibit collagen production by liver and lung fibroblasts [105]. In a mouse model of SSc, the production of profibrotic cytokines, such as IL-4, IL-6, IL-17, and TGF-β1, was attenuated by rapamycin. In addition, sirolimus treatment inhibited proliferation and collagen production of mouse fibroblasts in a dose-dependent manner [106].

In a small pilot study, sirolimus and MTX were compared in SSc patients. Rapamycin was well tolerated but the disease activity scores at 48 weeks and the changes in these scores from baseline were not significantly different between the groups [107].

Another phase II study of sirolimus treatment in SSc was planned and registered but the current status is unknown (NCT03365869).

Tofacitinib is an oral Janus kinase (JAK)-1/3 inhibitor that is currently approved for the treatment of rheumatoid arthritis, psoriatic arthritis, and ulcerative colitis. JAKs are intracellular enzymes that phosphorylate and activate Signal Transducers and Activators of Transcription (STATs), which in turn modulate intracellular activity including gene expression. Tofacitinib modulates the signaling pathway at the point of JAKs, preventing the phosphorylation and activation of STATs, and the production of several proinflammatory cytokines [108].

A recent study showed that JAK/STAT gene signatures were aberrant in biopsies from SSc patients, as well as JAK and STAT3 phosphorylation in both skin and lung biopsies. Furthermore, treatment of mice with the selective JAK inhibitor tofacitinib not only prevented bleomycin-induced skin and lung fibrosis but also improved established skin fibrosis [109].

Given its potential antifibrotic effect, tofacitinib has been recently evaluated in a phase I/II study in SSc patients. Among 15 participants, tofacitinib was well tolerated and there were trends in efficacy favoring tofacitinib at month 6, including the skin score. Other non-randomized studies suggested the potential efficacy of tofacitinib particularly for cutaneous involvement [110]. A phase III trial of tofacitinib for diffuse SSc has not been planned yet.

Subcutaneous and intravenous immunoglobulins (Ig) are approved for the treatment of various autoimmune diseases, including chronic inflammatory demyelinating polyneuropathy (CIDP) and immune thrombocytopenic purpura (ITP). The mechanisms of action of Ig are complex, but are mostly due to different pathways that depend on the Fc and/or the F(ab′)2 fragments [111]. The off-label use of Ig in patients with SSc provided evidence of their potential efficacy on multiple clinical manifestations, including skin fibrosis, gastrointestinal involvement, muscle strength and quality of life [112,113]. A phase II trial investigating the safety and efficacy of both subcutaneous and intravenous Ig in SSc is currently ongoing (NCT04137224).

Brentuximab vedotin is an anti-CD30 monoclonal antibody, currently approved for the treatment of anaplastic large cell lymphoma, cutaneous T-cell lymphoma, Hodgkin’s disease, and mycosis fungoides. Following binding to CD30, brentuximab vedotin is rapidly internalized and transported to lysosomes where monomethyl auristatin E (MMAE) is released and binds to tubulin, leading to cell cycle arrest and apoptosis [114]. A small pilot study that will treat ten patients with early or active dcSSc with brentuximab vedotin is ongoing (NCT03198689).

Bermekimab is a human IgG1 monoclonal antibody that neutralizes the activity of human IL-1α. IL-1α is up-regulated in the lesional skin and serum of SSc patients and also induces the production of IL-6 and PDGF, promoting the fibrosis [115].

A phase II proof-of-concept trial (NCT04045743) of bermekimab administration in 20 patients with SSc has just been completed.

Oncostatin M (OSM) is a pleiotropic member of the glycoprotein 130 (gp130)/IL-6 cytokine family that also includes IL-6 [116]. It is produced by leukocytes, including macrophages, activated T cells and neutrophils, and acts primarily via OSM receptors on a broad range of cell types. OSM functions include activation of endothelium, induction of the acute phase response, induction of cellular proliferation and/or differentiation of cell types such as fibroblasts, epithelial cells and keratinocytes, modulation of erythropoiesis and megakaryopoiesis, inflammatory mediator release, and promotion of wound healing. OSM has been implicated in a broad range of inflammatory and fibrotic diseases, including SSc [117].

GSK2330811 is a humanized immunoglobulin G1 kappa (IgG1κ) monoclonal antibody that functionally blocks human OSM from binding to the gp130 receptor. A randomized and placebo-controlled phase II trial enrolling 35 SSc patients has recently been completed (NCT03041025) and the results awaited.

Ziritaxestat (GLPG 1690) is an autotaxin inhibitor that has been recently evaluated in a pilot phase II study in SSc patients. Autotaxin was shown to be required for the development and maintenance of dermal fibrosis in the bleomycin mouse model of SSc, enabling two major mediators of fibrogenesis, lysophosphatidic acid (LPA) and IL-6, to amplify each other’s production [118]. Additionally, pharmacologic inhibition of autotaxin attenuated dermal fibrosis and IL-6 expression in the mouse model. Moreover, autotaxin was found to be overexpressed in skin samples from SSc patients [119]. Increased autotaxin levels were also observed in pulmonary fibrosis, where it was found to contribute to the activation of TGF signaling and the stimulation of fibroblast accumulation [120]. In the small phase II NOVESA study, ziritaxestat significantly improved the skin score versus placebo at week 24 and was well tolerated (NCT03798366). These results suggest that inhibition of the autotaxin pathway may be an effective therapeutic strategy for SSc.

Belumosudil (KD 025) is an inhibitor of Rho-associated coiled-coil kinase 2 (ROCK2). It binds to and inhibits the serine/threonine kinase activity of ROCK2, downregulating its signaling pathways, which play major roles in pro-and anti-inflammatory immune cell responses [121].

Increased ROCK activity has been found in the lungs of patients with IPF and treatment with belumosudil reduced lung fibrosis in the bleomycin mouse model [122]. Therefore, belumosudil may have a therapeutic benefit by targeting the fibrotic processes mediated by the ROCK signaling pathway.

Basing on these promising results, a phase II clinical trial of belumosudil administration in patients with diffuse SSc is currently ongoing (NCT03919799). In this trial, a total of 60 adult subjects will be enrolled and randomized into three groups to either receive orally administered belumosudil (two doses) or matched placebo for 28 weeks. The study will be double-blinded for the first 28 weeks followed by an open label extension period of 24 weeks. Another open-label study of belumosudil in diffuse cutaneous SSc has been recently initiated (NCT04680975).

Dersimelagon (MT-7117) is a selective melanocortin-1 receptor (MC1R) agonist that is currently being evaluated in a phase II study in SSc (NCT04440592). α-Melanocytic Hormone, the ligand of MC1R, has been implicated in chronic inflammation and fibrosis and evaluated in various preclinical models, including the bleomycin-induced fibrosis [123]. It is thought to exert its mechanism of action by reducing proinflammatory and profibrotic mediators such as TGF-β.

### 4.3. Agents in Phase I or Supported by Preclinical Evidence

TGF-β is a master regulator a of the pathogenesis of SSc. Various processes, including cell growth, apoptosis, cell differentiation, and extracellular matrix synthesis are regulated by TGF-β, a type of cytokine secreted by macrophages and many other cell types [124].

AVID200 is a novel inhibitor of TGF-β1 and β3, avoiding TGF-β2 related cardiac and hematopoietic toxicity. Overexpression of these TGF-β isoforms has been closely associated with the progression of fibrosis and cancer [125]. A Phase Ib trial of AVID 200 administration in SSc patients has just been completed (NCT03831438). Preliminary results demonstrated anti-fibrotic effects of AVID200 as indicated by rapid and sustained declines in skin fibrosis. AVID200 was well-tolerated, and no dose-limiting toxicities were observed [126].

ACE-1334 is another TGF-βRII/IgG1 fusion protein that inhibits TGF-β1 and 3, but not TGF-β2. It has shown robust anti-fibrotic activity in multiple preclinical models of fibrosis, and a phase I/II trial in SSc is set to start soon (NCT04948554).

FT-011 is a novel antifibrotic that, in vitro and in preclinical models, inhibited both TGF-β1 and PDGF-BB induced collagen production. A small phase II clinical trial to evaluate its effect in SSc is about to start (NCT04647890).

TEPEZZA (teprotumumab-trbw, HZN-001) is a fully human monoclonal antibody inhibitor of the IGF-1 receptor (IGF-1R). While being an important survival factor for various cell types, IGF-1 has also been implicated in fibrotic disorders, including SSc, where serum IGF-1 levels are elevated in patients with more severe skin and lung fibrosis [127,128]. A phase I trial of TEPEZZA in patients with diffuse cutaneous SSc is ongoing (NCT04478994).

Ifetroban is a potent and selective thomboxane A2/prostaglandin H2 receptor antagonist. TxA2 and its precursors are thought to play an important role in vascular contraction and have been implicated in platelet activation, as well as vasculo-inflammatory activation leading to the characteristic pathologies of chronic ulcers [129]. A proof-of-concept clinical trial of ifetroban administration in SSc is currently recruiting (NCT02682511).

Mesenchymal stromal cells (MSC) are adult multipotent cells that can be isolated from bone marrow (BM), adipose tissue, umbilical cord (UC), and other tissues, and, moreover, their ability of differentiating into different cell lineages have raised growing interest because of their broad immunomodulatory properties. Preclinical evidence has suggested their potential efficacy in fibrotic disorders. For example, systemic administration of human UC-MSC significantly reduced lung inflammation and fibrosis in bleomycin mouse models through a selective inhibition of the IL6-IL10–TGFβ axis involving lung M2 macrophages [33]. Phase I studies and single case reports also suggested that MSC are well tolerated and potentially effective in patients with SSc [130]. A small proof-of-concept study evaluating the effect of UC-MSC infusion in SSc patients is set to star this year (NCT04356287).

Lysophosphatidic acid (LPA) is a phospholipid growth factor that targets specific G-protein-coupled receptors that could possibly contribute to excessive tissue fibrosis, primarily through the activation of the LPA 1 receptor [131].

SAR100842 is a low molecular weight, orally available selective inhibitor of LPA 1 receptor that has been evaluated in a small study in SSc [132]. There was a clinically important skin score improvement after 24 weeks of treatment, and the drug was overall well tolerated, with mild to moderate adverse effects. The clinical development program of SAR100842 in SSc was initially discontinued by the manufacturer, but a phase II study of this compound, now renamed HZN-825, has recently started enrollment (NCT04781543).

Anifrolumab is a human IgG1*κ* monoclonal antibody that has been tested in a phase I trial to treat SSc [133]. This drug blocks the formation of the ternary IFN/IFNAR1/IFNAR2 signaling complex by sterically inhibiting the binding of IFN ligands to IFNAR1 [134]. Anifrolumab has recently shown to be effective in SLE in a phase III trial [135], whereas the clinical development for SSc has been discontinued by the manufacturer.

Basiliximab is a chimeric monoclonal antibody directed against the α chain (CD25) of the IL-2 receptor that has recently been proposed for the treatment of SSc based on the latest discoveries regarding the crucial role of effector T cells in this disease, particularly Th17 and T regulatory subsets. In an open-label study [136], basiliximab was well tolerated in SSc patients, showing potential efficacy on skin thickness, but no trials in SSc have been planned yet.

Otherwise known as hyperimmune caprine serum, AIMSPRO is a polyclonal antibody that contains mainly caprine immunoglobulins as well as cytokines, including IL-4 and IL-10, proopiomelanocortin, arginine vasopressin, β-endorphin, and corticotropin-releasing factor. In a phase II double-blind placebo-controlled trial AIMSPRO treatment was well tolerated and showed potential efficacy on skin thickness [137].

As previously mentioned, TLR4 stimulation promotes the production of cytokines by Th1 and Th17 cells, and increased levels of this molecule and its ligands have been found in SSc patients. TAK242, a small molecule TLR4 inhibitor, was studied in preclinical fibrosis models. This treatment prevented, and promoted the regression of, bleomycin-induced dermal and pulmonary fibrosis, and reduced the expression of several pro-fibrotic mediators. Furthermore, TAK242 reduced spontaneous hypodermal thickness in the model mice and abrogated collagen synthesis and myofibroblasts differentiation in explanted constitutively active SSc fibroblast [138]. To date, no trials in SSc patients with TAK242 have been planned yet.

Imatinib is a TKI that blocks both PDGF and TGF-β signaling pathways. It showed antifibrotic effects in SSc experimental models [139]. Dasatinib and nilotinib, which are second-generation TKIs, were also evaluated for the treatment of dermal fibrosis in vitro and in murine models with promising results [140].

In a phase II pilot study, imatinib had no significant effects on skin involvement in SSc patients, but appeared to stabilize lung function in patients with SSc-ILD [141]. However, no phase III trial in SSc has been conducted or planned.

Fresolimumab is a monoclonal antibody that can target all isoforms of TGFβ and has yielded very promising results in SSc [142]. Patients treated with this drug experienced a rapid improvement in skin scores, as well as a downregulation of the expression of TGFβ-regulated genes. However, no further trials in SSc have been planned yet.

Abituzumab is a novel, humanized monoclonal IgG2 antibody targeting CD51 (integrin alpha V), preventing ECM attachment, cell motility, and apoptosis, without cross-reacting with other integrins, which is elemental in inhibiting TGF-β [143]. Unfortunately, the phase III trial in SSc that had been planned was recently terminated due to difficulties in enrolling subjects under the eligibility criteria, not allowing for completion of the study within a reasonable time-frame.

## 5. Conclusions

Systemic sclerosis is an immune-mediated disease of unknown etiology, currently devoid of definitive therapy. Thanks to recent advances in deciphering some of the pathogenetic mechanisms, especially in the complex regulation of inflammatory and fibrotic processes, new therapeutic approaches are being evaluated in clinical trials, with the goal of hopefully changing the natural history of this high-impact multi-organ disorder.

## Figures and Tables

**Table 1:** Autoantibodies in systemic sclerosis.

Antibody	ANA Staining Pattern	Target	Clinical Associations
Anti-centromere (ACA)	Discrete speckled	CENP-A, B and C	lcSSc, PAH, peripheral vascular disease
Anti-topoisomerase I (Anti-SCl-70)	Speckled	DNA topoisomerase I	dcSSc, ILD
Anti-RNA polymerase III	Speckled	RNA polymerase III complex	dcSSc, rapidly progressive skin thickening, renal crisis, malignancy, GAVE
Anti-U3 RNP	Nucleolar	U3 RNP complex	ILD, PAH, renal crisis, small bowel disease
Anti-Th/To	Nucleolar	RNase P and RNase MRP	lcSSc, ILD, PAH
Anti-PmScl	Nucleolar	Exosome protein complex (PM-Scl-100, PM-Scl-75)	SSc-myositis overlap
Anti-U1 RNP	Speckled	U1 RNP complex	Inflammatory arthritis, myositis, PAH (MCTD)
Anti-Ku	Speckled	Ku80 and Ku70	Myositis
Anti-RuvBL1/2	Speckled	RuvBL1 and RuvBL2	dcSSc, myositis
Anti-Ro52	Speckled	TRIM21	ILD, overlap syndromes
Anti-Nor90	Nucleolar	Nucleolar transcription factor 1	Unclear (favourable prognosis?)
Anti-ANP32A	N/D	Acidic leucine-rich nuclear phosphoprotein	PAH

**Table 2:** Currently available treatment options for SSc.

Drug	Route of Administration	Mechanism of Action	Indication
Nifedipin	Oral	Calcium channel blocker	RP prevention
Iloprost	Intravenous	Synthetic analogue of prostacyclin PGI_2_	RP prevention; digital ulcers
Fluoxetine	Oral	SSRI	RP prevention
Sildenafil	Oral	PDE5 inhibitor	PAH Refractory RP Digital ulcers
Tadalafil	Oral	PDE5 inhibitor	PAH Digital ulcers
Bosentan	Oral	Endothelin receptor antagonist	PAH Digital ulcers
Ambrisentan	Oral	Endothelin receptor antagonist	PAH
Macitentan	Oral	Endothelin receptor antagonist	PAH
Riociguat	Oral	Stimulator of soluble guanylate cyclase	PAH
Selexipag	Oral	Prostacyclin receptor agonist	PAH
ACE inhibitors	Oral	Inhibition of ACE	Renal crisis
Methotrexate	Oral, subcutaneous	Dihydrofolate reductase inhibitor	Early diffuse skin disease Arthritis
Cyclophosphamide	Oral or intravenous	Precursor of an alkylating nitrogen mustard	Progressive skin disease ILD
Mycophenolate mofetil	Oral	Inhibitor of inosine monophosphate dehydrogenase	Progressive skin disease ILD
Autologous HSCT	/	Resets the immune system	Rapidly progressive disease
Abatacept	Subcutaneous	CD80/86-CD28 costimulatory pathway inhibitor	Progressive skin disease
Rituximab	Intravenous	Anti-CD20	Progressive skin disease ILD
Nintedanib	Oral	Tyrosine kinase inhibitor	ILD
Tocilizumab	Subcutaneous	IL-6R inhibitor	ILD Arthritis

Abbreviations: PDE: phosphodiesterase; PGI2: prostaglandin I2; PAH: pulmonary arterial hypertension; ILD: interstitial lung disease; HSCT: hematopoietic stem cell transplantation; ACE: angiotensin-converting enzyme; RP: Raynaud’s phenomenon; SSRI: selective serotonin reuptake inhibitor.

**Table 3:** Ongoing or recently completed pharmacological clinical trials in systemic sclerosis.

Drug	Mechanism of Action	Trial Identifier	Phase	Sample Size	Estimated or Actual Completion Date
Romilkimab	Bispecific IL4/IL13 antibody	NCT02921971	II	97	April 2019
Sirolimus	mTOR inhibitor	NCT03365869	II	72	June 2019
Tofacitinib	Janus kinase inhibitor	NCT03274076	I/II	15	November 2019
GSK 2330811	Oncostatin M inhibitor	NCT03041025	II	35	July 2020
Ziritaxestat	Autotaxin inhibitor	NCT03798366	II	33	June 2020
Bortezomib	Proteasome inhibitor	NCT02370693	II	30	June 2020
Lenabasum	Cannabinoid receptor CB2 agonist	NCT03398837	III	365	December 2020
AVID 200	TGF-β inhibitor	NCT03831438	Ib	24	January 2021
Bermekimab	IL-1 alpha inhibitor	NCT04045743	II	20	July 2021
IgPro20/IgPro10	Human normal immunoglobulin	NCT04137224	II	26	August 2021
Belimumab + rituximab	Anti-BAFF Anti-CD20	NCT03844061	II	30	February 2022
FT-011	TGF-ß1/PDGF-BB inhibitor	NCT04647890	II	30	April 2022
Belumosudil	Rho-associated kinase inhibitor	NCT03919799	II	60	May 2022
Pirfenidone + MMF	Antifibrotic	NCT03856853	II	150	June 2022
Brentuximab Vedotin	Anti-CD30	NCT03198689	II	10	July 2022
TEPEZZA	IGF-1R inhibitor	NCT04478994	II	25	September 2022
Guselkumab	IL-23p40 inhibitor	NCT04683029	II	56	December 2022
Ifetroban	TxA2/PGH2 receptor inhibitor	NCT02682511	II	34	December 2022
hUC-MSC	Umbilical cord derived MSC	NCT04356287	I/II	18	December 2022
Dersimelagon	Melanocortin type I agonist	NCT04440592	II	72	February 2023
Brodalumab	IL-17 receptor antagonist	NCT03957681	III	100	March 2023
HZN-825	LPA receptor inhibitor	NCT04781543	II	300	July 2023
Ixazomib	Proteasome inhibitor	NCT04837131	II	12	April 2024
ACE-1334	TGF-βRII/IgG1 fusion protein	NCT04948554	I/II	210	May 2028

Abbreviations: mTOR: mammalian target of rapamycin; TGF: transforming growth factor; BAFF: B-Cell Activating Factor; PDGF: Platelet-Derived Growth Factor; MMF: Mycophenolate mofetil; IGF: insulin-like growth factor; TxA2/PGH2: thromboxane A2/prostaglandin H2; MSC: mesenchymal stromal cell; LPA: Lysophosphatidic acid.

## Data Availability

No new data were created or analyzed in this study. Data sharing is not applicable to this article.
